# The filamentous phage XacF1 causes loss of virulence in *Xanthomonas axonopodis* pv. *citri*, the causative agent of citrus canker disease

**DOI:** 10.3389/fmicb.2014.00321

**Published:** 2014-07-01

**Authors:** Abdelmonim Ali Ahmad, Ahmed Askora, Takeru Kawasaki, Makoto Fujie, Takashi Yamada

**Affiliations:** ^1^Department of Molecular Biotechnology, Graduate School of Advanced Sciences of Matter, Hiroshima UniversityHigashi-Hiroshima, Japan; ^2^Department of Microbiology, Faculty of Science, Zagazig UniversityZagazig, Sharkia, Egypt

**Keywords:** filamentous phage, loss of virulence, citrus canker, biocontrol

## Abstract

In this study, filamentous phage XacF1, which can infect *Xanthomonas axonopodis* pv. *citri* (*Xac*) strains, was isolated and characterized. Electron microscopy showed that XacF1 is a member of the family *Inoviridae* and is about 600 nm long. The genome of XacF1 is 7325 nucleotides in size, containing 13 predicted open reading frames (ORFs), some of which showed significant homology to Ff-like phage proteins such as ORF1 (pII), ORF2 (pV), ORF6 (pIII), and ORF8 (pVI). XacF1 showed a relatively wide host range, infecting seven out of 11 strains tested in this study. Frequently, XacF1 was found to be integrated into the genome of *Xac* strains. This integration occurred at the host *dif* site (*att*B) and was mediated by the host XerC/D recombination system. The *att*P sequence was identical to that of *Xanthomonas* phage Cf1c. Interestingly, infection by XacF1 phage caused several physiological changes to the bacterial host cells, including lower levels of extracellular polysaccharide production, reduced motility, slower growth rate, and a dramatic reduction in virulence. In particular, the reduction in virulence suggested possible utilization of XacF1 as a biological control agent against citrus canker disease.

## Introduction

*Xanthomonas axonopodis* pv. *citri, Xac* (syn. *Xanthomonas campestris* pv. *citri*), is the causative agent of Asiatic citrus canker disease (ACC), one of the most serious citrus plant diseases in the world (Civerolo, 1984; Graham et al., 2004). Under natural conditions, the bacterium can invade all aboveground parts of plants, entering through natural openings and wounds (Brunings and Gabriel, 2003; Vojnov et al., 2010). A characteristic symptoms include raised corky lesions surrounded by a water or oil-soaked margin on leaves, stems, and fruits, including defoliation, twigs dieback, general tree decline, blemished fruit, and premature fruit drop in severely infected trees (Graham et al., 2004). Management of ACC relies on an integrated approach that includes: (1) replacement of susceptible citrus species with resistant ones; (2) production of disease-free nursery stock; (3) reduction of pathogen spread by establishing windbreaks and fences around groves; (4) preventative copper sprays; and (5) application of insecticide to control Asian leafminer. Because of the limited effectiveness of the current integrated management strategies, citrus canker disease continues to be an economically serious problem for field-grown crops worldwide (Balogh et al., 2010). Hence, alternative control methods are necessary.

Bacteriophages have recently been evaluated for controlling a number of phytobacteria and are now commercially available for some diseases (Balogh et al., 2010). The use of phages for disease control is a fast expanding area of plant protection, with great potential to replace existing chemical control measures. Bacteriophages have been used effectively for controlling several diseases caused by *Xanthomonas* species, including, peach bacterial spot, caused by *X. campestris* pv. *pruni*, geranium bacterial blight, caused by *X. campestris* pv. *pelargonii*, tomato bacterial spot caused by *Xanthomonas euvesicatoria* and *Xanthomonas perforans*, and onion leaf blight caused by *X. axonopodis* pv. *allii* (Flaherty et al., 2000; Balogh et al., 2003; Obradovic et al., 2004, 2005; Lang et al., 2007). Major challenges of agricultural use of phages arise from the inherent diversity of target bacteria, high probability of resistance development, and weak phage persistence in the plant environment (Balogh et al., 2008, 2010). Very recently, utilization of filamentous phages as a disease management strategy has been investigated, and application will likely increase in the future (Askora et al., 2009; Addy et al., 2012). The filamentous ϕRSM phages have dramatic effects on the virulence of *Ralstonia solanacearum*. Infection of *R. solanacearum* cells with ϕRSM3 decreased their growth rate, twitching motility, movement in tomato plant stems, extracellular polysaccharide (EPS) production, and *phcA* expression, resulting in loss of virulence (Addy et al., 2012). This strategy using filamentous phage might be expanded to control various diseases, including citrus canker disease. In contrast to lytic phages, filamentous phages do not kill the host cells but establish a persistent association between the host and the phage (Askora et al., 2009; Addy et al., 2012). This is an advantage of filamentous phages to solve the problem of bacteriophages easily inactivated by sunlight UV irradiation (Balogh et al., 2010).

In the current study, we isolated and characterized a novel filamentous phage and showed that changes occurred at a cellular level in *X. axonopodis* pv. *citri* strains following infection. This filamentous phage might be a unique biological agent for use against bacterial citrus canker disease.

## Materials and methods

### Bacterial strains and growth conditions

Ministry of Agriculture, Forestry, and Fisheries (MAFF) strains of *X. axonopodis* pv. *citri, Xac* (Table 1) were obtained from the National Institute of Agrobiological Sciences, Japan. Strain KC33 was obtained from the National Institute of Fruit Tree Science, the National Agriculture and Food Research Organization, Japan. All strains were stored at −80°C in 0.8% nutrient broth (NB) (BBL, Becton Dickinson and Co., Cockeysville, MD, USA) supplemented with 30% (v/v) glycerol. The strains were grown on nutrient agar (NA) medium (Difco, BBLBD, Cockeysville, MD, USA) at 28°C. For preparation of bacterial suspension, *Xac* strains were cultured for 24 h at 28°C with shaking at 220 rpm in NB medium.

**Table 1:** **Bacterial strains used in this study^a^**.

**Strain**	**Host (Citrus species)**	**XacF1 sensitivity**	**Source**
***X.axonopodis* pv. *citri***			
MAFF 301077	*C. limon*	−	NIAS^b^
MAFF 301080	*C. sinensis*	+	NIAS
MAFF 311130	*C. iyo*	−	NIAS
MAFF 302102	*Citrus* sp.	+	NIAS
MAFF 673001	*C. natsudaidai*	+	NIAS
MAFF 673010	*Citrus* sp.	+	NIAS
MAFF 673011	*C. limon*	−	NIAS
MAFF 673013	*Citrus* sp.	+	NIAS
MAFF 673018	*Citrus* sp.	+	NIAS
MAFF 673021	*C. limon*	−	NIAS
KC33	*C. iyo*	+	Shiotani et al., 2007
Phages			
XacF1			This study

a *All strains originated in Japan*.

b *NIAS, National Institute of Agrobiological sciences, Japan*.

For time course experiments, phage-infected and uninfected cells were grown overnight in 5 mL of NB media. Then, 0.5 mL of the cell suspensions (10^8^ cfu/mL) were transferred into 100-mL flasks containing 30 mL of NB medium. Cultures were grown at 28°C with agitation at 200 rpm, and OD_600 nm_ measurements were taken every 3 h over the course of 48 h using a spectrophotometer. Three replicates were included at each time point. The experiments were repeated twice (Li and Wang, 2011).

### Bacteriophage isolation, purification, and characterization

The presence of filamentous phages in collected soil samples from cropping fields in Japan was detected by the spot test and plaque-forming assay technique (Yamada et al., 2007). Approximately 10 g of soil was placed in a sterile 50 mL conical centrifuge tube that then was filled to the top with tap water, and allowed to stand for 20 min with periodic inversions. The tubes were then centrifuged at 15,000 × g for 20 min and the supernatant was passed through a membrane filter (0.45-μ m pore size) (Millipore Corp., Bedford, MA, USA). One-hundred-microliter aliquots of the soil filtrate were subjected to spot test and plaque-forming assay with strains of *Xac* (Table 1) as host on NB plates containing 1.5% (w/v) agar. Phages were propagated and purified from single-plaque isolates. An overnight culture of bacteria grown in NB medium (1 mL) was diluted 100-fold with 100 mL of fresh NB medium in a 500 mL flask. To collect a sufficient amount of phage particles, a total of 2 L of bacterial culture was grown. When the cultures reached an OD_600_ of 0.2, bacteriophage was added at a multiplicity of infection (moi) of 0.001–1.0. After further growth for 12–24 h, the cells were removed by centrifugation in a Hitachi Himac CR21E centrifuge with an R12A2 rotor at 8000 × g for 15 min at 4°C. The supernatant was passed through a 0.45-μ m-pore membrane filter followed by precipitation of the phage particles in the presence of 0.5 M NaCl and 5% (v/v) polyethylene glycol 6000 (Kanto Chemical Co., Tokyo, Japan). The pellet was collected by centrifugation in a Hitachi Himac CR21E centrifuge with an RPR20-2 rotor at 15 000 × g for 30 min at 4°C, and was dissolved in SM buffer [50 mM Tris/HCl at pH 7.5, 100 mM NaCl, 10 mM MgSO_4_ and 0.01% gelatin (w/v)]. Phages were stored at 4°C in complete darkness. Phage titers were determined by serial dilution and subsequent plaque-forming assays (Yamada et al., 2007). The purified phage [10^13^ pfu/mL was stained with sodium phosphotungstate prior to observation in a Hitachi H600A electron microscope, according to the methods of Dykstra (1993)].

### Phage susceptibility and adsorption assays

The phage susceptibility assays were based on a standard agar overlay method with dilution series of phage preparations (Yamada et al., 2007; Ahmad et al., 2014). Small turbid plaques, typical of Ff-phages, always appeared at reasonable frequencies depending on input phage titers (usually 300–600 pfu/plate), if the bacterial strain was sensitive to the phage. No spontaneous phages (induced prophages) appeared from either strain tested under usual plaque assay conditions. In the phage adsorption assay, exponentially growing cells (OD_600_ 0.1) of the test strain were mixed with XacF1 phage at moi of 0.1, and the mixture was incubated for 0 min (no adsorption) and 30 min at 28°C to allow binding of the phage to the cell surface. Following centrifugation at 15,000 × g for 5 min at 4°C in a Sakuma SS-1500 microcentrifuge (Sakuma Seisakusho, Tokyo, Japan), the phage titer in the supernatant was determined by a standard plaque assay with the indicator strain (MAFF301080). *Escherichia coli* JM109 was used as a negative control.

### DNA isolation and manipulation

Standard molecular biological techniques for DNA isolation, digestion with restriction enzymes and other nucleases, and construction of recombinant DNAs were followed, according to Sambrook and Russell (2001). Phage DNA was isolated from the purified phage particles by phenol extraction. In some cases, extrachromosomal DNA was isolated from phage-infected *Xac* cells by the minipreparation method (Ausubel et al., 1995). Replicative-form (RF) DNA for sequencing was isolated from host bacterial cells infected with XacF1 phage, treated with S1 nuclease, and then shotgun-sequenced by Hokkaido System Science Co. (Sapporo, Japan) using a Roche GS Junior Sequence System. The draft assembly of the obtained sequences was assembled using GS *De novo* Assembler v2.6. The analyzed sequences corresponded to 156 times the final genome size of XacF1 (7325 bp). Computer-aided analysis of the nucleotide sequence data was performed using DNASIS v3.6 (Hitachi Software Engineering Co., Tokyo, Japan). Potential ORFs larger than 80 bp were identified using the online program ORF Finder (http://www.ncbi.nlm.nih.gov/gorf/gorf.html) and the DNASIS program. Sequence alignment was performed using the ClustalW (Larkin et al., 2007) program. To assign possible functions to the ORFs, DDBJ/EMBL/GenBank databases were searched using the FASTA, FASTX, BLASTN, and BLASTX programs (Altschul et al., 1997).

### Determination of *att*L and *att*R sequences in *xac* MAFF673010

Chromosomal DNA was extracted from *Xac* MAFF673010 after infection with XacF1 and subjected to PCR to amplify fragments containing left and right attachment sites (*att*L and *att*R). The *att*L was amplified using a 29-base forward primer, 5′-TGC GAT CGA GCA GCT TCC CAG TTG GCG AT-3′ (primer P1) and a 30-base reverse primer, 5′-TTC GAT GGT CAC GGT GCC TGT AGT AGA GGC-3′ (primer P2), while *att*R was amplified using a 30-base forward primer, 5′-ATA ATT TGC TTG ACA CCG TGC GCA AGT CGT 3′ (primer P3) and a 28-base reverse primer, 5′-CCT TGA CCG TCA GGG ACT GCA TCA GCC T-3′ (primer P4). The primer sequences were based on the *dif* (*att*B) region sequence of *Xanthomonas citri* subsp. citri Aw12879 (DDBJ accession no. CP003778.1). The PCR products were purified from an agarose gel and subjected to sequencing.

### Southern hybridization

Genomic DNA from bacterial cells was prepared by the minipreparation method according to Ausubel et al. (1995). Following digestion with restriction enzyme *Hin*cII, DNA fragments were separated by agarose gel electrophoresis, blotted onto a nylon membrane (Piodyne; Pall Gelman Laboratory, Closter, NJ, USA), hybridized with a probe (the entire XacF1 DNA digested by *Eco*RI), labeled with fluorescein (Gene Images Random Prime labeling kit; Amersham Biosciences, Uppsala, Sweden), and detected with a Gene Images CDP-Star detection module (Amersham Biosciences). Hybridization was performed in buffer containing 5× SSC (0.75 M NaCl, 0.075 M sodium citrate), 0.1% (w/v) sodium dodecyl sulfate (SDS), 5% liquid block, and 5% (w/v) dextran sulfate for 16 h at 65°C. The filter was washed at 60°C in 1× SSC and 0.1% (w/v) SDS for 15 min and then in 0.5× SSC and 0.1% (w/v) SDS for 15 min with agitation, according to the manufacturer's protocol. The hybridization signals were detected by exposing X-ray film (RX-U; Fuji Film, Tokyo, Japan) to the filter.

### EPS assay

EPS in bacterial culture supernatants was determined quantitatively as described previously (Guo et al., 2010). Briefly, bacterial strains were grown in NB supplemented with 2% (w/v) D-glucose for 24 h at 28°C with shaking at 200 rpm. A 10-mL portion of the culture was collected, and the cells were removed by centrifugation (5000 × g for 20 min). The supernatant was mixed with three volumes of 99% ethanol and the mixture was kept at 4°C for 30 min. To determine the dry weights of EPS, the precipitated EPS was collected by centrifugation and dried at 55°C overnight prior to measurement. Three replicates were used for each strain and the test was repeated three times.

### Motility assay

Swimming and swarming motilities were examined on NB containing 0.3% (w/v) and 0.7% (w/v) agar (Difco, Franklin Lakes, NJ, USA), respectively. Overnight cultures of bacteria grown in NB were centrifuged at 8000 × g for 2 min at 4°C, washed twice with ddH_2_O, and resuspended in ddH_2_O (OD_600_ = 1.0). Two microliters of the suspension were spotted onto NA plates (diameter, 90 mm; containing 20 mL of NA) and incubated at 28°C. The migration zones were measured, and used to evaluate the motility of *Xac* cells (Li and Wang, 2011; Addy et al., 2012). For twitching motility assays, overnight bacterial culture in NB were centrifuged at 8000 × g for 2 min at 4°C, washed twice with ddH_2_O, resuspended in ddH_2_O (OD_600_ = 1.0), and spotted on minimal medium (MM) plates (Addy et al., 2012). Plates were incubated at 28°C, and the morphology of the colony edge was observed under a light microscope (100× magnification).

### Pathogenicity assay

After careful washing with tap water, immature fully expanded lemon leaves were sterilized by soaking for 2 min in sodium hypochlorite, followed by rinsing in sterilized water. Leaves were placed on the surface of filter paper with abaxial surfaces facing upwards. Lemon leaves were inoculated with bacterial suspension of *Xac* phage-uninfected and phage-infected strains (10^8^ cfu/mL in sterile water) using an infiltration or a needle pricking method. The infiltration method was conducted by pushing a needleless syringe containing the bacterial suspension against the surface of a citrus leaf supported by a finger on the opposite side of the leaf. The treated areas were immediately marked following inoculation (Chen et al., 2012). Needle-prick inoculation was performed by pricking the leaves, and droplets (10 μ L) of bacterial suspensions were applied to each inoculation site. In both methods, the inoculated leaves were covered with a plastic bag for 48 h to facilitate the infection. Leaves were incubated in a growth chamber at 28°C with a photoperiod of 12 h light and 12 h dark for 4 weeks (Verniére et al., 1998; Li and Wang, 2011; Malamud et al., 2012).

### Phage stability test

We used *Xac* strain MAFF301080 because it was free from a XacF1 sequence in the genome. After infection with XacF1, a single colony was isolated and confirmed for its production of phage particles, the presence of XacF1 DNA in the cells by miniprep, and no integration of XacF1 DNA in the chromosome by PCR with a primer set of chromosomal sequences, 5′-ACT CGC TTT GCA TGA AAT TCG CTA GCG AT-3′ (forward) and 5′-TTC GAT GGT CAC GGT GCC TGT AGT AGA GGC (reverse). After cultivation in NB at 28°C for several generations, random colonies spread on NA plates were picked and subjected to plaque assay, miniprep for XacF1 DNA, and PCR to detect lysogeny with the same primers as above.

#### Nucleotide sequence accession number

The sequence of the XacF1 genome has been deposited in the DDBJ under accession no. AB910602.

## Results

### Isolation, morphology, and host range of xacF1

**Figure 1:**
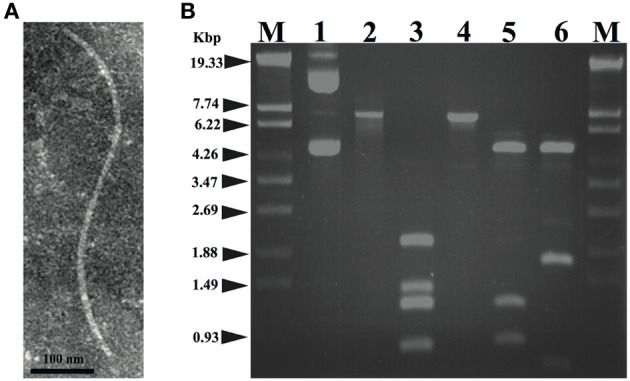
**(A)** Morphology of the XacF1 phage. The purified particles of XacF1 were negatively stained with phosphotungstate and examined by transmission electron microscopy. A filamentous structure was observed (approximately 600 nm in length). **(B)** Restriction patterns of the replicative form of the XacF1 genomic DNA. Lanes: 1, undigested XacF1 DNA (RF); 2, digested with S1 nuclease; 3, *Hin*cII; 4, *Eco*RI; 5, *Eco*RV; 6, *Cla*I; M, λ *Sty*I marker.

A total of 20 phages were isolated from soil samples collected from citrus fields in Japan using a plaque assay on *Xac* strains (see Experimental Procedures), one of which formed small and turbid plaques (designated XacF1). A single plaque of this phage was picked for propagation, purification, and further experiments. Electron micrographs using highly purified phage particles (10^13^ pfu/mL) showed that XacF1 virions have typical filamentous phage features, with a long fibrous shape approximately 600 nm in length (Figure 1A). To determine the host range of the phage, *Xac* strains infecting different citrus species were tested for phage susceptibility (Table 1). The host range of the XacF1 phage was relatively wide, infecting 7 out of 11 *Xac* strains tested in this study (Table 1).

### Nucleotide sequence and genomic organization of xacF1

The genomic DNA of XacF1 was obtained as a replicative form (RF) from MAFF301080 as a host. XacF1 phage genomic DNA was digested using several restriction enzymes; *Eco*RI digestion produced a single band corresponding to approximately 7.3 kb on an agarose gel (Figure 1B, lane 4). The genomic DNA isolated from phage particles was completely digested by S1 nuclease treatment (data not shown), suggesting that the XacF1 genome is a circular single stranded DNA, like those of all other filamentous phages.

**Figure 2:**
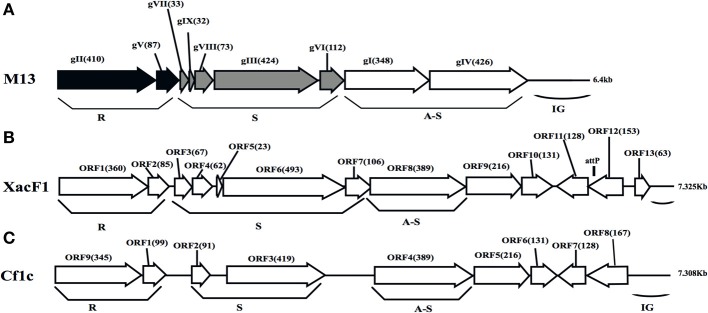
**Genomic organization of bacteriophage XacF1**. Linear genomic maps of *E. coli* phage M13 **(A)**, XacF1 **(B)**, and Cf1c **(C)** are compared. Arrows oriented in the direction of transcription represent ORFs or genes. The functional modules for replication (R), structure (S), and assembly-secretion (A-S) are indicated according to the M13 model (Marvin, 1998). Map for Cf1c was drawn according to the genomic sequence (accession no. NC_001396, Kuo et al., 1991). ORF sizes (in amino acids) are in parentheses. IG (intergenic region), and *att*P are also shown.

To determine the entire nucleotide sequence of XacF1, DNA was shotgun-sequenced. The results showed that the complete genome was 7325 nucleotides long, with a G+C content of 57.8%, which was significantly lower than that of the host genome (i.e., 64.7% for strain 306, accession no. NC_003919). There were 13 putative open reading frames (ORFs), of which 11 were located on the same strand and two were on the opposite strand (Table 2 and Figure 2). When databases were searched for sequences homologous to the XacF1 DNA sequence using BLAST and BLASTX programs, nine ORFs showed high similarity to ORFs previously reported for other filamentous phages, especially to ORFs of *X. campestris* pv. *citri* phage Cf1c (Kuo et al., 1991) (accession no. NC_001396), *X. campestris* pv. *vesicatoria* Cf1 phage (YP_364205.1), and *X. campestris* pv. *campestris* phi-Lf phage (X70328) (Table 2). XacF1 ORFs could be arranged in a similar modular structure to that of previously characterized filamentous phages of the Ff group (Model and Russel, 1988; Marvin, 1998), as shown in Figure 2. Within the putative replication module (Figure 2), we identified ORF1 and ORF2. The peptide encoded by ORF1 was homologous to filamentous phage phi-Lf replication initiation protein II (98% amino acid sequence identity) (Table 2). This gene encodes the pII protein, which is necessary for rolling-circle replication of phage genomes (Model and Russel, 1988). The deduced amino acid sequence encoded by ORF2 was homologous to peptides that mapped at the same position as the ssDNA binding protein (gV gene) of Ff phages, and its size was similar to that of this binding protein (Figure 2 and Table 2). Within the putative structural module of XacF1, we predicted five ORFs. ORF3 showed similarity to a hypothetical *Xanthomonas* protein (Table 2), with 32% amino acid sequence identity to a transmembrane motif (WP_005416529), supporting the hypothesis that ORF3 belongs to the module of structural genes (Figure 2). Moreover, ORF4, ORF5, and ORF7 (Figure 2 and Table 2) were the same size and in the same position as genes encoding the coat proteins of Ff phages. Another possible ORF included in this module was ORF6 (with similarity to coat protein Cf1c phage cp3, Kuo et al., 1991), which was similar in both size and location to *gIII* of the Ff phage. *gIII* encodes *p*III, a minor coat protein that recognizes and interacts with receptors and coreceptors on the host cells (Armstrong et al., 1981; Lubkowski et al., 1999; Heilpern and Waldor, 2003) (Figure 2 and Table 2). It also showed 28% amino acid sequence identity to phage adsorption protein of *Xanthomonas citri* subsp. *citri* (YP_007649573). Therefore, ORF6 could be a homolog of g*III* in XacF1. In the third putative module of XacF1, the assembly module, we found that ORF8 showed the highest homology to the cp4 protein of Cf1c phage (Figure 2 and Table 2), and to the zot protein of *Xanthomonas vesicatoria* (WP_005997731), with 59% amino acid sequence identity. Also, based on its size and position, it seems that ORF8 is a homolog of *p*I. XacF1 does not encode a *p*IV homolog, hence like many filamentous phages it must use a host encoded *p*IV homolog, outer membrane protein of the secretin family. Interestingly, we found that ORF12 might encode a regulator gene similar to those found in several filamentous phages, because amino acids encoded by this ORF exhibited similarity to several putative transcriptional regulators and DNA-binding helix-turn-helix proteins of phages (e.g., Cp8 of *X. campestris* pv. *citri* phage Cf1c (99% amino acid identity) (Shieh et al., 1991); phage repressor of *Vibrio parahaemolyticus* V-223/04, exhibiting 45% amino acid identity, EVU16279, *E*-value = 0.71). ORFs 9, 10, 11, and 13 had homology to hypothetical proteins of phages and bacteria, but did not appear to belong to any of the previously described modules.

**Table 2:** **Predicted ORFs found in the XacF1 genome**.

**Coding sequence**	**Strand**	**Position 5′–3′**	**GC content (%)**	**Length of protein (aa)**	**Molecular mass (Kda)**	**Amino acid sequence identity/similarity to best homologs (no. of amino acid identical; % identity)**	***E*-value**	**Accession no**.
ORF1	+	1–1080	57.7	360	40.5	Filamentous phage phiLf replication initiation protein II (340; 98)	0.0	YP_005637352
ORF2	+	1077–1373	57.3	99	9.1	V protein *Xanthomonas* phage Cf1c (73; 99)	2e-46	NP_536673
ORF3	+	1405–1605	51.4	67	7.2	Hypothetical protein- *Xanthomonas* (65; 98)	2e-40	WP_010378728
ORF4	+	1611–1868	60.5	62	8.4	B coat protein- *Xanthomonas* phage Cf1c (62; 100)	6e-33	Q38618
ORF5	+	1928–1995	55	23	5.9	No significant similarity	–	
ORF6	+	1996–3474	55.8	493	51.7	A coat protein- *Xanthomonas* phage Cf1c (383; 96)	0.0	Q38619
ORF7	+	3474–3791	54.2	106	11.5	Hypothetical protein- *Xanthomonas campestris* (103; 98)	5e-67	WP_010378725
ORF8	+	3788–4954	59.0	389	42.8	Hypothetical protein Cf1cp4- *Xanthomonas* phage Cf1c (388; 100)	0.0	NP_040477
ORF9	+	4954–5601	59.4	216	23.5	Hypothetical protein Cf1cp5- *Xanthomonas* phage Cf1c (214; 99)	4e-148	NP_536676
ORF10	+	5617–6009	56.6	131	14.4	Hypothetical protein Cf1cp6- *Xanthomonas* phage Cf1c (130; 100)	4e-88	NP_536677
ORF11	−	6047–6430	59.7	128	14.4	Hypothetical protein Cf1cp7- *Xanthomonas* phage Cf1c (127; 100)	1e-86	NP_536678
ORF12	−	6427–6885	57.6	153	16.4	- Filamentous phage Cf1 protein- *Xanthomonas campestris* pv. *vesicatoria* str. 85-10 (146; 90) - 18.2K protein- *Xanthomonas* phage Cf1c (164; 99)	3e-88 5e-113	YP_364205 NP_536679
ORF13	+	7015–7203	54.8	63	6.8	Hypothetical protein- *Xanthomonas axonopodis* (59; 95)	6e-32	WP_017171337

### XacF1 uses host xerCD recombinases to integrate into the *xanthomonas* genome

**Figure 3:**
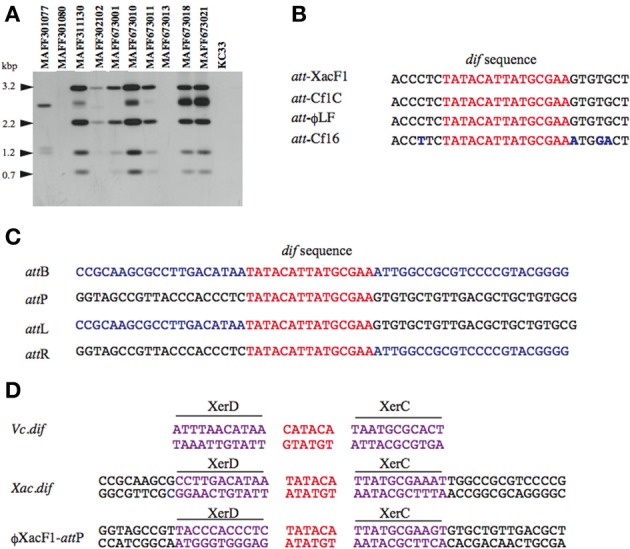
**Site specific integration of XacF1 DNA into the chromosome of *Xac*. (A)** Southern blot hybridization analysis showing integration of the XacF1 phage into *Xac* host chromosomes. Genomic DNA of *X. axonopodis* pv. *citri* strains was digested with *Hin*cII and hybridized with a probe (the entire XacF1 phage genomic DNA digested with *Eco*RI). **(B)** The *att*P sequence indicated for XacF1 is compared with *att*P sequences (putative in some cases) of filamentous phages, including Cf1c (accession no. NC_001396), Cf16-v1 (M23621), and Lf (X70328) phages of *X. campestris*. The *dif* core sequences are shown in red. Changed nucleotides are shown in blue. **(C)** Alignment of *att* sequences in the XacF1 integration system. The *att*L and *att*R sequences were determined for a lysogenic strain newly established with *Xac* MAFF673010 infected with XacF1. The *att*B sequence (*dif*) of *X. axonopodis* pv. *citri* 306 was obtained from the genome database (accession no. AE008923.1, Jalan et al., 2011). Common 15 bases are shown in red and chromosomal sequences are in blue. **(D)** Putative XerC, XerD binding sites of the XacF1 system are compared with those of *Vibrio cholerae dif*1.

Homology searches of the DDBJ/EMBL/GenBank databases for the XacF1 sequence revealed similar sequences in the genomes of some *Xanthomonas* species. This result suggested possible integration of this kind of phage into the host genome. To test this possibility, we performed genomic Southern blot analysis of 11 strains of *Xac* using a XacF1 DNA probe. The results, shown in Figure 3A, indicated that eight of the 11 strains contained hybridizing bands and, among them, seven strains showed similar hybridization patterns with variations in signal intensity. Therefore, XacF1 likely has a lysogenic cycle and integrates frequently into the host genome. Regarding the integration mechanism of XacF1, we could not find any genes or ORFs that encode a phage integrase in the genome (Table 2). In several cases, involvement of the host recombination system by XerC/D in integration of filamentous phages into host genomes has been established, including *Vibrio cholerae* phage CTXϕ (Huber and Waldor, 2002; Das et al., 2011). In CTXϕ integration, the *dif* site of the host genome (*att*B) forms a recombination complex with *dif*-like sequences on the phage genome (*att*P) (Val et al., 2005). We therefore looked for a possible *dif*-like sequence for *att*P on the XacF1 sequence and found a 15-bp *dif* core sequence of 5′-TAT ACA TTA TGC GAA (XacF1 positions 6504–6518). This sequence showed a high degree of homology to *att*P sequences of phages Cf1c (accession no. NC_001396) (Kuo et al., 1991), Cf16-v1 (M23621), ϕLf (X70328), CTXϕ (AΦ220606), and ϕVGJ (AY242528) (Figure 3B). It was also reported that Cf1c, Cf1t, Cf16v1, and ϕLf phages of *X. campestris* use the XerCD recombinases of their host to integrate into the *dif* locus of the bacterial genome (Campos et al., 2003; de Mello Varani et al., 2008; Askora et al., 2012; Das et al., 2013). These results suggested that the filamentous phage XacF1 uses the host XerC/D system for integration into the host genome. To confirm this, we obtained both *att*L and *att*R fragments by PCR from newly established XacF1-lysogenic cells of *X. axonopodis* pv. *citri* strain MAFF673010. The *att*L and *att*R sequences are aligned with XacF1 *att*P and *dif* of *X. axonopodis* pv. *citri* strain 306 (accession no. AE008923.1, Jalan et al., 2011) in Figure 3C. From these results, we predicted XerCD binding sites according to Das et al. (2011) as shown in Figure 3D. However, XacF1 *att*P is located within the coding region of ORF12, so following integration into *att*B of the host chromosome, ORF12 may be split into two portions. This change in ORF12 may affect XacF1 functions because ORF12 encodes a possible phage regulator, as described above.

### Effects of xacF1 infection on the growth rate of *x. axonopodis* pv. *citri*

**Figure 4:**
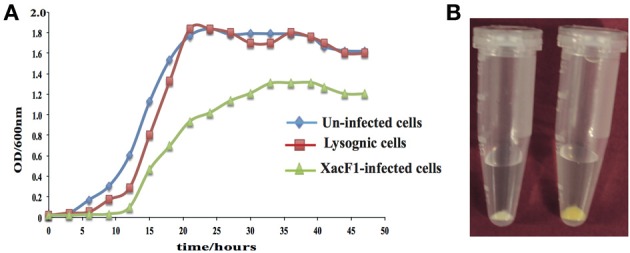
**(A)** Effects of XacF1 infection on the growth of *X. axonopodis* pv. *citri* (MAFF301080). **(B)** Effects of XacF1 infection on EPS production. The pellet of uninfected *Xac* MAFF301080 cells (right) was yellow, while the pellet of XacF1-infected cells (left) was white, indicating a defect in xanthan production.

Unlike other bacterial viruses, the Ff phages do not kill their hosts, but establish a persistent coexistence in which new virions are continually released (Model and Russel, 1988). Because of this non-lytic mode of viral replication, it is possible to grow high-titer cultures of the virus. Similarly, infection by XacF1 did not cause lysis of host cells, but established a persistent association between the host and phage, releasing phage particles from the growing host cells. Although cells infected with XacF1 could continue to grow and divide indefinitely, the process caused the infected cells to grow at a significantly lower rate than uninfected cells (Figure 4A).

### Effect of xacF1 infection on host EPS production

EPS production was compared between uninfected and XacF1-infected cells of strain MAFF301080. The XacF1-infected cells used in this experiment were confirmed to be free from prophage by plaque assay of the culture supernatant, Southern hybridization (Figure 3A), and PCR. The amount of EPS produced by the infected cells was significantly lower than that of the wild-type cells. We observed that following centrifugation, the culture pellets of the infected cells turned white, reflecting a low production of xanthan, which is the major component of EPS and is responsible for the yellow color of *Xanthomona*s culture in the media (Figure 4B). Our prediction was confirmed by an EPS quantitation assay, which showed that the XacF1-infected cells had significantly lower EPS production (0.6 mg/ 10^10^ cfu) than uninfected cells (3.35 mg/ 10^10^ cfu).

### Effect of xacF1 infection on host motility

**Figure 5:**
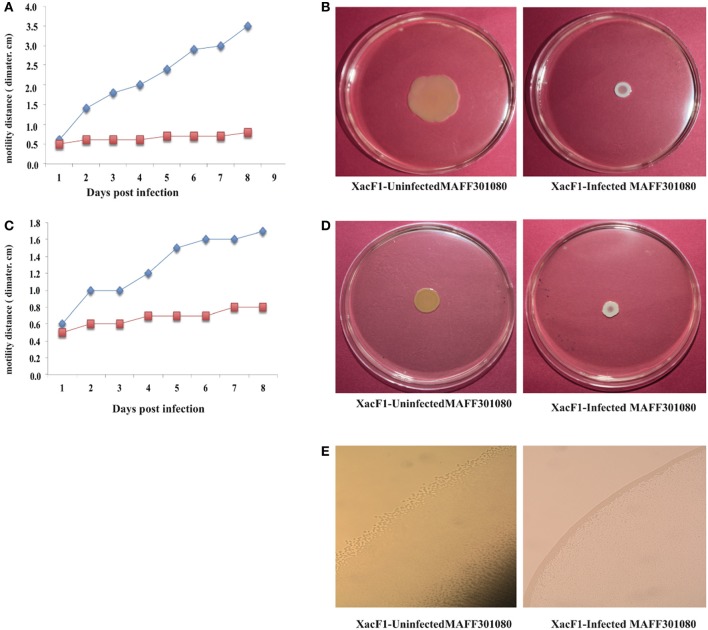
**Impact of XacF1 infection on the motility of *Xac* MAFF301080 cells**. Two microliters of bacterial solution [10^8^ colony forming units (CFU)/mL] were inoculated in the swimming assay [0.3% (w/v) agar] **(A,B)**, swarming assay [0.7% (w/v) agar] **(C,D)**, and twitching motility assay (minimal agar medium) **(E)**. The movement of bacterial cells was photographed 5 and 8 days post-inoculation (dpi) on the swimming and swarming plates, respectively. Twitching motility of bacteria was observed under a microscope 5 dpi on the twitching plates.

Swimming, swarming, and twitching motilities of uninfected and XacF1-infected cells of strain MAFF301080 were compared. A significant reduction in swimming and swarming motility was observed in XacF1-infected cells (Figures 5A–D). When visualized with a microscope, the colony margin of uninfected cells had a highly irregular shape, indicating proficient twitching motility, whereas the colony edge of XacF1-infected cells was smooth (Figure 5E), suggesting a decrease or loss of twitching motility. Because twitching motility is the surface movement associated with type IV pili (Marques et al., 2002; Meng et al., 2005), XacF1 infection may have affected the type IV pilus structures and/or functions of the host cells. We examined whether cell surface structural components were affected by XacF1 infection. Cell surface structure proteins were prepared by passing bacterial cells through a hypodermic needle, separated by SDS-PAGE, and compared between XacF1-infected and uninfected cells. XacF1-infected cells had considerably decreased levels of PilA, the major component of type IV pili, and decreased levels of FilC, flagellin (Supplemental Figure S1).

### Effects of xacF1 infection on virulence of *x. axonopodis* pv. *citri*

**Figure 6:**
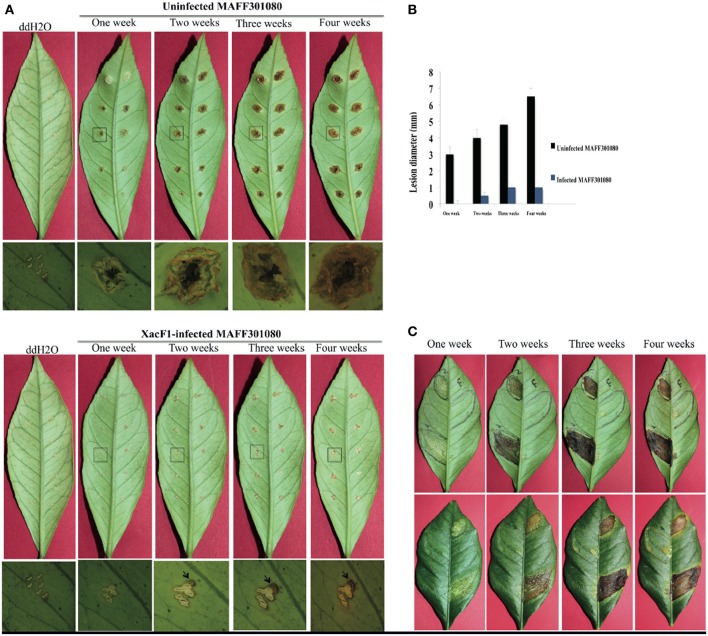
**Lesions on detached lemon leaves inoculated with cells of *Xac* MAFF301080. (A)** Canker symptoms that had developed on leaves 1, 2, 3, and 4 weeks post-infection by the needle-pricking method. Leaves were inoculated with uninfected cells (upper panels) or XacF1-infected cells (lower panels). Leaf areas shown by a square were examined by photomicroscopy and the microscopic images are shown under each corresponding leaf. Characteristic canker lesions occurred with uninfected cells, while no obvious cankers developed on XacF1-infected cells. **(B)** Comparison of the size of lesions formed on lemon leaves. **(C)** Lesions formed on lemon leaves by infiltration of bacterial cells. Uninfected MAFF301080 cells were applied to two areas of the leaf (left half of the abaxial side), and XacF1-infected cells were similarly applied to the right side (upper panels). Lesions on the axial side are also shown in lower panels. Lesions on both lower and upper surfaces of leaves inoculated with the uninfected cells showed severe symptoms, expanding with time. No lesions formed on either surface of the leaves infected with XacF1-infected cells.

Wild-type cells of strain MAFF301080 caused infection symptoms 4 days post-infection, and formed clear cankers 1 week post-inoculation (Figure 6A). Starting from 2 weeks post-infection, the lesion became brown in color and its center became raised and spongy or corky, typical canker symptoms (Graham et al., 2004) that reflected the aggressive virulence of this strain. In contrast, the symptoms of XacF1-infected MAFF301080 cells were relatively weak, and no mature canker symptoms were observed up to 4 weeks post-infection, except for marginal lesions formed around the pricking site (Figure 6A). To be more precise, we measured lesion size (Figure 6B), which showed that in uninfected cells, the lesions were large with a smooth center, spongy raised top, and their distribution around the infected area reached more than 6.5 mm in width 4 weeks post-infection. In contrast, the lesions formed by XacF1-infected cells remained weak and dry, and they did not expand more than 1 mm in width. Another inoculation method, in which we infiltrated the bacterial suspension into the lemon leaves, showed that XacF1-uninfected MAFF301080 cells incurred water soaking at the inoculation site 3 days post-infection, and then an erumpent tissue reaction was obvious 1 week after inoculation. The erumpent tissue expanded to an aggressive canker area on both sides of the leaf, and then the lesions became dark and decayed with a yellow halo at the inoculation site 4 weeks post-infection (Figure 6C). However, in XacF1-infected cells, a slight water-soaking area on the leaf surface was only visible 2 weeks after inoculation, and weak canker symptoms could be seen 4 weeks post-infection. In all cases, leaves inoculated with ddH_2_O showed no canker symptoms.

## Discussion

In this study, we isolated and characterized a filamentous phage, named XacF1, that infects *X. axonopodis* pv. *citri* strains. The isolated phage had a relatively wide range of host bacterial strains. Of particular interest, this study showed that along with the phage infection, the infected cells had decreased ability to form citrus cankers and a loss of virulence. We demonstrated that the canker symptoms of XacF1-infected lemon leaves were dramatically mitigated up to 4 weeks post-infection using both pricking and infiltration methods of inoculation (Figures 6A–C). The significant reduction in EPS (xanthan) production caused by XacF1 phage could be one of the reasons for such a dramatic decrease in canker formation. Virulence of numerous phytopathogenic bacteria, particularly various *Xanthomonas* species, is correlated with their ability to produce EPS (Dolph et al., 1988; Bellemann and Geider, 1992; Kao et al., 1992; Chou et al., 1997; Katzen et al., 1998; Dharmapuri and Sonti, 1999; Yu et al., 1999; Kemp et al., 2004). The multiple functions of EPS in virulence include protection of bacteria from toxic plant compounds, reduction of bacterial contact with plant cells to minimize host defense responses, promotion of bacterial multiplication by prolonging water soaking of tissues, and supporting invasion or systemic colonization of bacterial cells (Denny, 1999). Another possible role of EPS is to confer epiphytic fitness. It was previously suggested that EPS functions during both epiphytic and pathogenic phases of infection in *X. campestris* pv. *campestris* (Poplawsky and Chun, 1998; Rigano et al., 2007). As significant differences in virulence were observed between wild-type and xanthan-deficient mutant strains of other *Xanthomonas* species, Dunger et al. (2007) proposed that in citrus canker, xanthan supports epiphytic survival in citrus canker, but is not required for colonizing nearby tissue. Without xanthan the bacteria were unable to retain water and could not withstand abiotic stress and, thus, could not survive on the leaf surface. Therefore, xanthan works in two ways: to enhance bacterial virulence and to block the host defense. The drastic reduction in host EPS production caused by XacF1 infection may explain why the XacF1-infected cells showed dramatically decreased virulence.

Another major finding is the significant reduction in the swimming, swarming, and twitching motilities of *Xac* cells following infection by XacF1. Bacteria use a variety of motility mechanisms to colonize host tissues. These mechanisms include flagella-dependent swimming and swarming for movement in liquid surfaces, and flagella-independent twitching, gliding, and sliding for movement on solid surfaces (O'Toole and Kolter, 1998; Mattick, 2002; Harshey, 2003). Recent reports propose that bacterial adhesion and motility are required in the initial stages of *Xac* biofilm formation, whereas lipopolysaccharide and EPS play important roles in the establishment of mature biofilms (Li and Wang, 2011). The reduction in motility of XacF1-infected cells may be because filamentous phages such as XacF1 assemble on the host cell membrane and protrude from the cell surface, and so the nature of the host cell surface may change drastically during phage production (Addy et al., 2012). As shown in Supplemental Figure S1, XacF1-infected cells had considerably decreased levels of PilA, the major component of type IV pili.

Frequent protrusion of XacF1 particles from the infected cell surface may somehow compete with the formation of type four pili (Tfp). As reported by Kang et al. (2002), Tfp is responsible for twitching motility and adherence to multiple surfaces and is required for virulence. Interestingly, ORF 9 of the XacF1 phage (Table 2) showed significant homology to a TraX family protein (H8FlE6, *E*-value = 1e-70) and a putative F pilin acetylation protein (Q3BsT0, *E*-value = 4e-70), involved in pilus modification. Therefore, the loss of virulence in the XacF1-infected cells seems to be, at least partly, caused by the reduction or modification of Tfp formation and decrease in swimming, swarming, and twitching motilities.

Several works have described the use of phages for control of bacterial citrus canker caused by *X. campestris* pv. *citri* (Balogh et al., 2010). In those cases, the bacteriophages used for foliar plant diseases interacted with the target bacteria on the leaf surface, the phylloplane. The phylloplane is a constantly changing environment: there are changes in temperature, sunlight irradiation, leaf moisture, relative humidity, osmotic pressure, pH, microbial flora, and, in the case of agricultural plants, chemical compounds (Jones et al., 2012). These factors may be harmful to bacteriophages to varying extents. Sunlight irradiation, especially in the UVA and -B range, is mainly responsible for eliminating bacteriophages within hours of application (Jones et al., 2012). To avoid quick inactivation of XacF1, we propose the application of XacF1-infected cells instead of XacF1 phage alone. The XacF1-infected cells can grow and continue to produce infectious phage, so the XacF1 phage may serve as an efficient long-lasting tool to control citrus canker by decreasing the virulence of the pathogen. Concerning the stability of XacF1-infected cells, we observed relatively high stability of “a free phage state” in the infected cells. After several bacterial generations, 100% cells contained XacF1 and more than 70% of them were at the state of producing free phages without integration into the host chromosome (confirmed by PCR) (data not shown). Even if once prophage states were established, we observed frequent spontaneous excision and production of phage particles.

Another possible way to use XacF1 for biological control may be given as a phage cocktail with other lytic phages, such as Cp1 and Cp2, originating from Japan, which can infect more than 97% of *Xac* strains and was recently characterized by Ahmad et al. (2014).

### Conflict of interest statement

The authors declare that the research was conducted in the absence of any commercial or financial relationships that could be construed as a potential conflict of interest.
