# Supplementary material for: The filamentous phage XacF1 causes loss of virulence in Xanthomonas axonopodis pv. citri, the causative agent of citrus canker disease
Source: Front Microbiol. 2014 Jul 1;5:321. doi: 10.3389/fmicb.2014.00321 (PMC4076744; doi:10.3389/fmicb.2014.00321)
Supplement: Supplementary file 1 [file Presentation1.PDF]

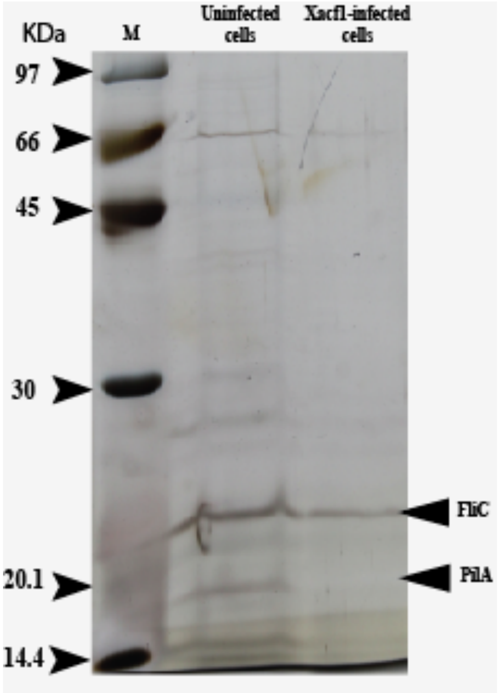

Supplemental Fig. S1. Comparison of proteins from cell surface structures. Cell surface appendages were released by passing bacterial cells through a hypodermic needle and their protein components were solubilized, separated by sodium dodecyl sulfate polyacrylamide gel electrophoresis, and stained with Coomassie blue. Molecular size of each marker protein (from Amersham LMW gel filtration kit) is indicated on the left. FliC, PilA, proteins were identified by their N-terminal amino acid sequence (Addy *et al*., 2012).

Methodology:

Cells of *Xac* MAFF301080 were streaked heavily onto MM plates and incubated for 48 h. The colonies were suspended in a small volume of 10 mM Tris-HCl buffer at pH 8, and the cell suspension (same cell density in each sample) was forced five times through a 25- gauge hypodermic needle (Clough *et al.*, 1994). Bacterial cells were removed by centrifugation at 8,000 × *g* for 20 min at 4°C. The bacterial surface appendages were collected by centrifugation at 136,000 × *g* for 60 min. Precipitated materials were subjected to Tris-Tricine sodium dodecyl sulfate polyacrylamide gel electrophoresis (SDS- PAGE) according to Schagger and von Jagow (Schagger *et al*., 1987). The identification of PilA was done according to Addy *et al*. (2012)

Clough, S.J., Schell, M.A., and Denny, T.P. (1994). Evidence for involvement of a volatile extracellular factor in *Pseudomonas solanacearum* virulence gene expression. *Mol. Plant-Microbe Interact.* 7, 621-630.

Schagger, H., and von Jagow, G. (1987). Tricine-sodium dodecyl sulfate-polyacrylamide gel electrophoresis for the separation of proteins in the range from 1 to 100 kDa. *Anal. Biochem.* 166, 368-379.
